# Carbon Storage Tanker Lifetime Assessment

**DOI:** 10.1002/gch2.202300011

**Published:** 2023-05-11

**Authors:** Oleg Gaidai, Qingsong Hu, Jingxiang Xu, Fang Wang, Yu Cao

**Affiliations:** ^1^ College of Engineering Science and Technology Shanghai Ocean University Shanghai 201306 China

**Keywords:** carbon capture and storage, CO_2_, energy, environment, subsea technology

## Abstract

CO_2_ capture and storage (CCS) is an important strategy to reduce global CO_2_ emissions. This work presents both cutting‐edge carbon storage tanker design, as well as novel reliability method making possible to extract useful information about the lifespan distribution of carbon capture systems from their recorded time history. The method outlined may be applied on more complex sustainable systems that are exposed to environmental stresses throughout the whole period of their planned service life. The latter is of paramount importance at the design stage for complex engineering systems. Novel design for CCS system is discussed and accurate numerical simulation results are used to apply suggested novel reliability methodology. Furthermore, traditional reliability approaches that deal with complex energy systems are not well suited for handling high dimensionality and cross‐correlation between various system components of innovative dynamic CO_2_ storage subsea shuttle tanker. This study has two distinctive key features: the state of art CCS design concept, and the novel general purpose reliability method, recently developed by authors, and particularly suitable for operational safety study of complex energy systems.

## Graphical abstract

The Intergovernmental Panel on Climate Change states that in order to prevent drastic effects of global warming, global greenhouse gas emissions must be cut. This study has two distinctive key features: the state of art carbon capture and storage design concept, and the novel general purpose reliability method, and particularly suitable for operational safety study of complex energy systems.

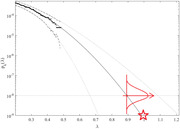

## 1 Introduction

**Figure 1:**
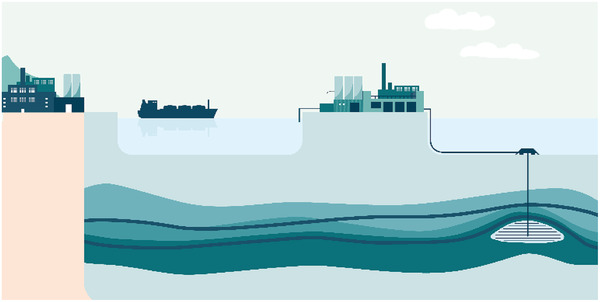
Equinor Northern lights carbon capture concept. Reproduced with permission.^[^ ^1^ ^]^ Copyright 2023, Equinor ASA.

One method of reducing climate change is carbon capture and storage (CCS). A significant portion of the Norwegian climate solution (NCS) will involve CCS. The Northern Lights project, which is a key component of the program the Norwegian government refers to as Longship and which has received investment from Equinor, Shell, and TotalEnergies, is Norway's first license for CO_2_ storage on the NCS, see **Figure** **1**.

The subsea shuttle tanker (SST) being novel Norwegian sizeable autonomous cargo submarine design, proposed by Equinor in 2019,^[^ ^1^ ^]^ and in 2020,^[^ ^2^ ^]^ SST baseline,^[^ ^3^ ^]^ being a 34 000 ton vessel, created to meet national carbon capture and storage (CCS) initiatives throughout the aquatic areas of the Norwegian Continental Shelf Sleipner, Snøhvit, Utgard.^[^ ^4^ ^]^ SST gathers CO_2_ from facilities off the Norwegian coast and then moves independently to the target subsea well for direct injection at a constant depth of 70 m below the water's surface. Because SST flows underwater and is unaffected by wave stresses, it can work in all weather. The major goal of SST is to develop as a replacement mode of transportation for remote, undeveloped areas once CO_2_ levels make ship tankers or offshore pipelines economically viable. SSTs considered in ref. [5] have been designed, following state‐of‐art DNV submarine design code.^[^ ^6^ ^]^ The use of design optimizations should increase the economic allure of SST.^[^ ^7^ ^]^ For SST baseline design parameters,^[^ ^3^ ^]^ see **Table** **1**. For alternative approaches on CO_2_ engineering research see refs. [8, 9]. Equinor has a novel suggestion for moving CO_2_ from the surface to the seabed: autonomous submarines. According to the Norwegian owner, the shuttles might also carry water and oil for injection.

**Table 1:** SST baseline design parameters

Parameter	Value	Units
Length	164	m
Beam	17	m
Weight	3.36 × 10^7^	kg
Center of buoyancy (CoB) [*x*_b_,*y*_b_,*z*_b_]	[0, 0, −0.41]	m
Skeg position *x*_s_	67	m
Skeg area *A*_S_	40	m^2^
Forward tunnel thruster position *x*_tf_	60	m
Aft tunnel thruster position *x*_ta_	−60	m
CO_2_ cargo capacity	1.7 × 10^6^	kg
Operating depth	70	m

For alternative approaches to CCS challenges, and associated numerical modeling solutions, see, e.g., recent works.^[^ ^10^, ^11^ ^]^

**Figure 2:**
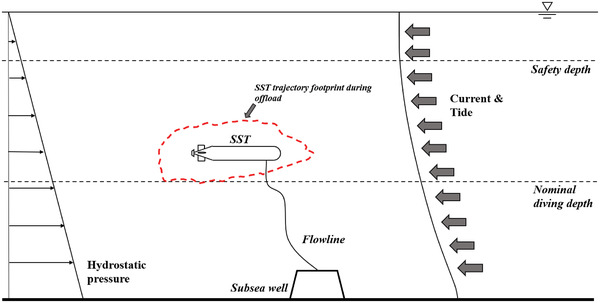
SST offloading while being exposed to in‐place environmental hydrodynamic loads. Reproduced with permission.^[^ ^12^ ^]^ Copyright 2021, ASME.

In order to dynamically maintain its position while being subject to in situ ambient hydrodynamic stresses from ocean subsurface currents, the SST vessel uses thrusters and a propeller, see **Figure** **2**.

It is crucial to comprehend severe postural reactions, such as excessive surge and heave. Extreme hydrostatic loads and substantial displacements experienced by SST during operation are referred as extreme heave movements. Hydrostatic force is frequently the main load that causes SST pressure hulls to collapse. The necessity to keep the flowline from getting taut necessitates excessive surge motions, which might result in severe snap loadings. The latter hazard becomes considerably more serious if the thrusters fail. SST might lose its maneuverability, resulting in obviously bigger responses. In this work, the aft thruster failure has been used as an illustration. In this research, an aft thruster failure scenario at SST during unloading is examined. This study employed empirical data from time‐domain simulations using a 2D planar Simulink model,^[^ ^13^, ^14^ ^]^ for further details see Section 2.

## 2 2D Planar SST Design

**Figure 3:**
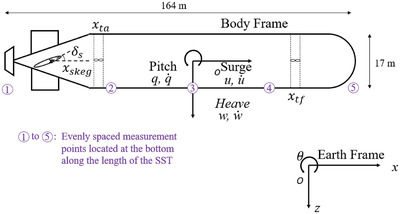
Coordinate system for SST with chosen measurement points. Reproduced with permission.^[^ ^12^ ^]^ Copyright 2021, ASME.

The SST center of gravity is located where the vehicle body‐fixed coordinate system is (CoG). Compared to an earth‐fixed reference frame, the motion of the body‐fixed reference frame moves (North, East, and Down). The SST CoG is placed directly over the CoB. The SST's geometric center is where the CoB is situated. In general terms, the coordinate system is stated in **Figure** **3**.

## 3 Experimental Section

**Figure 4:**
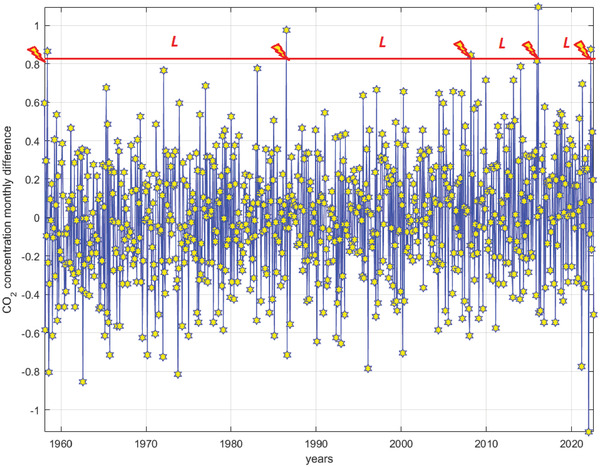
Illustration of consecutive lifetime distribution. Flashes indicate system failures.

This section presents methodology for lifetime distribution (LTD) estimation of complex SST dynamic systems, subjected to multiple failure modes during designed service time. **Figure** **4** represents lifetime distribution, corresponding to Mauna Loa Observatory monthly measured CO_2_ concentration consecutive differences.

Horizontal red line in Figure 4 indicates failure threshold, while consecutive time spans between data indicate crossing threshold, are denoted as *L*, illustrating environmental dynamic system consecutive lifetimes *L_i_*, *i* = 1, 2, …. Thus *L* being random process (variable), representing dynamic system lifetime.^[^ ^15^, ^16^, ^17^, ^18^, ^19^, ^20^, ^21^, ^22^ ^]^ In particular, when the number of system dimensions (failure modes) was high, modern engineering reliability methodologies did not provide a formula to analyze service level agreements of complex energy systems. In theory, it was eligible to assess target cumulative density function

(1) $$\begin{array}{c} \mathrm{LTD}\;\left(L\right)=\mathrm{Prob}\left(\mathrm{Lifetime}\leq L\right) \end{array}$$

an easy manner for a complicated environmental system, employing either enough measurement data or direct Monte Carlo simulations.^[^ ^23^, ^24^, ^25^, ^26^, ^27^, ^28^, ^29^ ^]^ Both the computing cost and the expense of experiments might be prohibitive for many complicated dynamic energy systems. In order to lower measurement and computation expenses during the system design phase, a unique lifespan evaluation technique for energy systems was created.

A structural dynamic multi‐degree of freedom (MDOF) response/load system vector, assembled of system components (*X*(*t*), *Y*(*t*), *Z*(*t*), …), being either measured or simulated over a sufficiently long (representative) time span (0, *T*) was taken into consideration. System component global maxima were then denoted as $X_{T}^{\max}=\max_{0\leq t\leq T}X\left(t\right)$, $Y_{T}^{\max}=\max_{0\leq t\leq T}Y\left(t\right)$, $Z_{T}^{\max}=\max_{0\leq t\leq T}Z\left(t\right),\text{…}$. By sufficiently long time span *T*, one meant large enough value of *T* with respect to environmental dynamic system auto‐correlation, as well as relaxation times. With $X_{1},\text{…},X_{N_{X}}$ being temporally consequent local maxima of the system, component process *X* = *X*(*t*) at discrete temporally increasing time instants $t_{1}^{X}<\text{…}<t_{N_{X}}^{X}$ within (0, *T*). Identical definitions were valid for other MDOF components: *Y*(*t*), *Z*(*t*), … namely, $Y_{1},\text{…},Y_{N_{Y}};$ $Z_{1},\text{…},Z_{N_{Z}}$ and so on. For simplicity, all system components were assumed to be nonnegative^[^ ^30^, ^31^, ^32^, ^33^ ^]^

(2) $$\begin{array}{c} P=\overset{\left(\eta_{X},\;\eta_{Y},\;\eta_{Z\;},\;\text{…}\right)}{\underset{\left(0,\;0,\;0,\;\text{…}\right)}{\;\int \int \int \;}}p_{X_{T}^{\max},\;Y_{T}^{\max},\;Z_{T}^{\max},\;\text{…}}\left(X_{T}^{\max},\;Y_{T}^{\max},\;Z_{T}^{\max},\;\text{…}\right)\;dX_{T}^{\max}dY_{N_{Y}}^{\max}dZ_{N_{z}}^{\max} \end{array}$$

**Figure 5:**
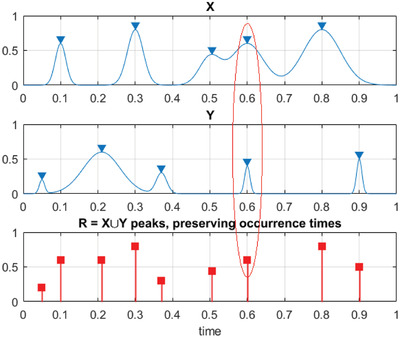
Example of how two components, X and Y, being merged, creating new synthetic vector $\vec{R}$. Red ellipse highlights case of simultaneous maxima for different components.

Being target probability of dynamic system survival, having critical values of system components denoted as *η*_*X*_, *η*_*Y*_, *η*_*Z*_,…; ∪ being logical unity operator «or»; and $p_{X_{T}^{\max},\;Y_{T}^{\max},\;Z_{T}^{\max},\;\text{…}}$ being joint probability density function (PDF) of individual component maxima. Is dynamic system number of degrees of freedom (NDOF) large, it might not be practically feasible to estimate directly joint PDF $p_{X_{T}^{\max},\;Y_{T}^{\max},\;Z_{T}^{\max},\;\text{…}}$ and thus target survival probability *P*. The latter target probability *P* needed to be estimated according to Equation (1)

(3) $$\begin{array}{c} L_{\mathrm{expected}}=E\;\left[L\right]=\int_{0}^{\infty }L\cdot \;\frac{T}{P_{\mathrm{failure}}}\mathrm{dLTD}\left(L\right) \end{array}$$

having failure probability *P*_failure_ = 1 − *P*, being complementary to a survival probability *P*. Dynamic system being regarded as immediately failed, if either its component *X*(*t*) exceeded *η*_*X*_, or *Y*(*t*) exceeded *η*_*Y*_, or *Z*(*t*) exceeded *η*_*Z*_, etc. Fixed system failure/hazard levels *η*_*X*_, *η*_*Y*_, *η*_*Z*_,… being individually set for each system unidimensional system component $X_{N_{X}}^{\max}=\max\;\left\{\;X_{j}\;;j=1,\text{…},N_{X}\right\}=X_{T}^{\max}$, $Y_{N_{Y}}^{\max}=\max\;\left\{\;Y_{j}\;;j=1,\text{…},N_{Y}\right\}=Y_{T}^{\max}$, $Z_{N_{z}}^{\max}=\max\;\left\{\;Z_{j}\;;j=1,\text{…},N_{Z}\right\}=Z_{T}^{\max}\;$, … Dynamic system unidimensional components *X*, *Y*, *Z*, … being re‐scaled and nondimensionalized

(4) $$\begin{array}{c} X\to \frac{X}{\lambda \eta_{X}},\;Y\to \frac{Y}{\lambda \eta_{Y}},\;Z\to \frac{X}{\lambda \eta_{X}} \end{array}$$

making all bio‐responses nondimensional and having the same target failure/hazard limit, when *λ*_fail_ = 1, with target failure/hazard probability *P* = *P*(1). Equation (2) might be used then to define *P*(*λ*) as a function of nondimensional level *λ*. Unidimensional dynamic system components local maxima, being merged into one temporally nondecreasing vector $R\left(t\right)\equiv \vec{R}=\left(R_{1},\;R_{2},\;\text{…},R_{N}\right)$ according to corresponding merged temporal vector *t*_1_ ≤ … ≤ *t_N_*, *N* ≤ *N_X_* + *N_Y_* + *N_Z_* + …. Each local maxima of *R_j_* being actual encountered dynamic system component local maxima, corresponding to either *X*(*t*) or *Y*(*t*), or *Z*(*t*), or other dynamic system components,^[^ ^34^, ^35^, ^36^, ^37^ ^]^ Constructed synthetic system $\vec{R}$‐vector thus having no data loss, see **Figure** **5**.

Having now introduced a nondecreasing synthetic vector $\vec{R}$, along with its corresponding temporally nondecreasing occurrence times *t*_1_ ≤ … ≤ *t_N_*, the lifetime stochastic process *L* = *L*(*t*) might be expressed as follows

(5) $$\begin{array}{c} L_{i}=t_{i}-t_{i-1} \end{array}$$

for *i* = 2, …, *N*. Thus composed synthetic process ***R*** (*t*) holds a key information about target LTD probability distribution. Survival probability *P* = *P*(1) might be expressed via mean up‐crossing rate function

(6) $$\begin{array}{c} P\left(\lambda \right)\approx \exp\;\left(-\nu^{+}\left(\lambda \right)\;T\right);\;\nu^{+}\left(\lambda \right)=\int_{0}^{\infty }\zeta p_{R\dot{R}}\left(\lambda ,\zeta \right)d\zeta \end{array}$$

with *ν* ^+^(*λ*) being mean up‐crossing rate of a failure/hazard threshold level *λ* for the above discussed synthetic nondimensional vector *R*(*t*). Mean up‐crossing rate function in Equation (6) was known as the Rice's with $p_{R\dot{R}}$ being joint PDF for $\left(R,\;\dot{R}\right)$ with $\dot{R}=R^{\prime }\left(t\right)$ being a time‐derivative. Dynamic system approached its designated failure limit *λ* → *λ*_fail_

(7) $$\begin{array}{c} \log_{\lambda \to 1}\;P_{\mathrm{failure}}=\frac{T}{L_{\mathrm{expected}}}=\nu^{+}\left(\lambda \right)\;T\;\Rightarrow \;L_{\mathrm{expected}}=\frac{1}{\nu^{+}\left(1\right)} \end{array}$$

according to Equation (3). MDOF dynamic energy system was assumed to be jointly‐stationary. Given in situ environmental scattered diagram having *m* = 1, .., *M* environmental states, each individual environmental short‐term environmental state had a probability *q_m_*, with $\sum_{m\;=\;1}^{M}q_{m}=1$. According to a long‐term probability equation

(8) $$\begin{array}{c} \mathrm{LTD}\left(L\right)\equiv \sum_{m\;=\;1}^{M}LTD_{m}\left(L\right)q_{m} \end{array}$$

with *p_k_* (*λ*,*m*) being same function as in Equation (7), corresponding to specific short‐term environmental sea state with number *m*. In the following section, one would illustrate how LTD *q* − quantiles of interest

(9) $$\begin{array}{c} L_{q}={\mathrm{LTD}}^{-1}\;\left(q\right) \end{array}$$

might be computed from available EH system measured/simulated underlying time series, with *q* ∈ (0, 1), and LTD^−1^ being an inverse function to the LTD, namely, LTD^○^ LTD^−1^ = 1, with 1 being identity operator. Note that at high failure levels, dynamic system failure events became independent, therefore lifetime distribution would follow Poisson distribution with parameter *ν* ^+^(*λ*) *T*. This study, although primary linked to the Poisson process concept, might have wider application, if system components reaching failure levels did not implicate imminent system failure, thus representing only series of alarming interdependent events.

## 4 Results

This section uses the SST hull displacements data set to demonstrate the effectiveness of the suggested approach. Naturally, the nonlinear, multidimensional, and cross‐correlated dynamics of SST multidimensional dynamic responses make them difficult to analyze. Five channels with simulated vertical displacement responses R_1_, …, R_5_ were chosen as components for the SST dynamic components in this investigation *X*, *Y*, … see Equation (2), Figure 3, forming an example of a 5D dynamic system. Simulated time series *X*, *Y*, *Z*, … have been scaled and nondimensionalized as follows in order to be combined according to Equation (4), resulting in the same nondimensional failure limit of 1. Then, by maintaining these five system components *X*, *Y*, … in temporally nondecreasing order, all local maxima from each measured time series were combined into a single time series $\vec{R}=\left(\max\left\{X_{1},Y_{1},\;\text{…}\right\},\text{…},\max\left\{X_{N},Y_{N},\;\text{…}\right\}\right)$. Note that advocated methodology may treat any number of dimensions, and 5D case here (namely, only five system components *X*, *Y*, …) has been chosen only as illustration.

**Figure 6:**
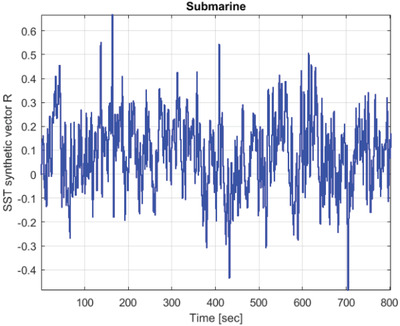
Example of SST displacements, presented as random time series.

**Figure** **6** presents an example of SST (submarine) vertical displacements, presented as stochastic time series.

**Figure 7:**
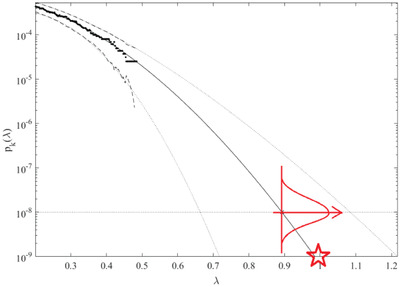
Extrapolation to the target level, star indicates target level with *λ*_fail_ = 1.

Dotted lines in **Figure** **7** highlight extrapolated 95% confidence interval (CI). Conditioning parameter *k* = 5 has been found to be sufficient, due to convergence with respect to *k*, namely, $\lim_{k\to \infty }p_{k}\left(\lambda \right)=P\left(\lambda \right)$, for proof see ref. [18]. Figure 7 exhibits reasonably narrow 95% CI, the latter being an advantage of the advocated approach. Failure/hazard levels *η*_*X*_, *η*_*Y*_, … were set equal to 50% higher than actual encountered component load/response maxima. Now, as soon as target failure level *λ*_fail_ up‐crossing rate *P*(*λ*) ≈ exp ( − *ν* ^+^(*λ*) *T*) have been extrapolated as in Figure 7, one may conclude that target dynamic system lifetime distribution will follow Poisson distribution with temporal parameter *ν* ^+^(1) , being system's failure rate, measured in years^−1^, having expected system lifetime $L_{\mathrm{expected}}=\frac{1}{\nu^{+}\left(1\right)}$ according to Equation (7).

## 5 Conclusions

The cross‐correlation between the responses of multiple energy systems and high‐dimensional systems cannot be handled by traditional time series reliability methodologies. The main advantage of the proposed method is its ability to study lifetime distribution of high‐dimensional nonlinear dynamic systems. This study looked at a carbon capture system dynamic behavior in arbitrary environmental conditions. Using a unique system reliability technique, the distribution of the device's service life throughout the period of its stipulated design lifespan was anticipated. The proposed strategy's theoretical foundation has undergone a thorough analysis. Due to their complexity and high dimensionality, dynamic systems demand the creation of novel, accurate, and trustworthy methodologies that can handle the available data and optimize its value, notwithstanding the appeal of using direct measurement or Monte Carlo simulation to evaluate the reliability of dynamic systems. Only for 1D system responses, the approach for this study has previously shown successful when used with a range of simulation models. Overall, forecasts were made, and they were extremely precise. The main goal of this work was to develop an all‐purpose, reliable, spatio‐temporal and user‐friendly multidimensional energy system reliability strategy.

This study has considered only vertical displacements of the CCS tanker, related to its dynamic motion control. Other environmental risks associated with CCS deep water operations, such as, e.g., extreme hull pressures, should be considered as well. Although authors have not studied dynamic pressure hull distribution, this study benchmarking novel reliability methodology that is well suitable for accounting of various complex in situ environmental loads.

As shown, the suggested method produced a fair confidence interval. The suggested approach may therefore prove valuable in a variety of reliability analyses of nonlinear dynamic systems. The implementation of fresh concepts is in no way constrained by the specified naval architecture example.

## Conflict of Interest

The authors declare no conflict of interest.

## Data Availability

The data that support the findings of this study are available from the corresponding author upon reasonable request.
